# Genome-wide association study of the fatty liver index in the Taiwanese population reveals shared and population-specific genetic risk factors across ethnicities

**DOI:** 10.1186/s13578-025-01346-5

**Published:** 2025-02-08

**Authors:** Pei Pei Lau, Chun-Yu Wei, Min-Rou Lin, Wan-Hsuan Chou, Yu-Jui Yvonne Wan, Wei-Chiao Chang

**Affiliations:** 1 Department of Clinical Pharmacy, School of Pharmacy, Taipei Medical University, Taipei, Taiwan; 2 Core Laboratory of Neoantigen Analysis for Personalized Cancer Vaccine, Office of R&D, Taipei Medical University, Taipei, Taiwan; 3 Department of Medical Pathology and Laboratory Medicine, University of California, Davis, Sacramento, CA USA; 4 Master Program in Clinical Genomics and Proteomics, School of Pharmacy, Taipei Medical University, Taipei, Taiwan; 5 Integrative Research Center for Critical Care, Wan Fang Hospital, Taipei Medical University, Taipei, Taiwan; 6 Department of Pharmacy, Wan Fang Hospital, Taipei Medical University, Taipei, Taiwan; 7 Department of Pharmacology, National Defense Medical Center, Taipei, Taiwan

**Keywords:** Fatty liver disease, Genome-wide association study, Fatty liver index

## Abstract

**Background and objectives:**

Although the incidence of fatty liver disease (FLD) is increasing worldwide, the genetic basis of this disease is not fully understood. This study uses the fatty liver index (FLI) to identify and compare genetic variants associated with FLD in Taiwanese and European populations.

**Results:**

In this study, a total of 145,356 Taiwan Biobank participants were included in the discovery analysis. Subjects with elevated FLI were found to have a significantly greater risk of developing FLD, as confirmed by imaging data (OR: 4.43; 95% CI: 3.88–5.06). Through genome-wide association studies (GWAS), we identified 6 variants previously associated with nonalcoholic fatty liver disease (NAFLD) and validated 50 shared risk variants located in *ZPR1* and *FTO* between the Taiwanese and European populations. Conditional analysis of 423 significant variants from FLI-defined FLD further revealed 16 independent variants within 14 genes. Pathway analysis of GWAS significant genes revealed that lipid metabolism and the peroxisome proliferator-activated receptor (PPAR) signaling pathway are causes of hepatic fat accumulation.

**Conclusion:**

This study identified six independent NAFLD-associated variants in *GCKR*, *LPL*, *TRIB1AL*, and *FTO* and emphasized *ZPR1* and *FTO* as shared risk genes for FLI-defined FLD in both Taiwanese and European populations. These findings support the utility of the FLI for FLD prediction, provide new genetic insights, and reveal the common genetic pathways of FLD across two ethnic groups. This research offers a valuable framework for advancing personalized medicine and therapeutic strategies for FLD.

**Supplementary Information:**

The online version contains supplementary material available at 10.1186/s13578-025-01346-5.

## Background

Fatty (steatotic) liver disease (FLD) is characterized by the accumulation of fat in the liver. This disease can be further classified etiologically into alcoholic-related liver disease (ALD), metabolic dysfunction-associated steatotic liver disease (MASLD), and metabolic dysfunction and alcohol-associated liver disease (MetALD) [1, 2]. MASLD has replaced the historical terms nonalcoholic fatty liver disease (NAFLD) and metabolic dysfunction-associated fatty liver disease (MAFLD) to reclassify FLD with inclusive metabolic criteria [1]. Without detection and management, FLD may gradually progress to hepatic fibrosis or cirrhosis, ultimately resulting in irreversible damage to the liver [3]. The global prevalence of MAFLD is reported to be 50.7% in overweight or obese individuals, with males exhibiting a higher prevalence than females at 59% versus 47.5% globally [4]. In Taiwan, 40.2% of the studied population is reported to have MAFLD, as reported with MAFLD in health examinations [5].

International experts suggest the diagnosis of FLD with blood biomarkers, radiologic imaging, or liver biopsy [6]. Among various imaging tests, ultrasound has limited sensitivity in detecting steatosis below 20% and hence is less accurate for individuals with a body mass index (BMI) greater than 40 kg/m^2^ [6]. Instead, for moderate to severe cases, computed tomography (CT) and magnetic resonance imaging (MRI) are preferred, although their high cost and need for specialized software limit their widespread application [6]. Moreover, liver biopsy, an invasive procedure, is reserved for patients with uninformative imaging and laboratory data [7]. Bedogni et al. proposed the fatty liver index (FLI) as an inexpensive and noninvasive surrogate to evaluate FLD through incorporating risk factors for FLD, such as waist circumference (WC), triglyceride (TG) level, BMI, and gamma-glutamyl transferase (GGT) level [8]. An FLI of 60 or higher is a reliable indicator of FLD, with a positive likelihood ratio of 4.3 [8].

In addition to alcohol consumption and metabolic factors, several genetic features attributable to FLD have been reported in previous studies [9]. After accounting for both genetic and environmental factors, the heritability estimates of NAFLD range from 20–50% [10]. Furthermore, Fairfield et al. performed a genome-wide association study (GWAS) using data from the UK Biobank and identified genes associated with NAFLD, such as *PNPLA3*, *TM6SF2*, and *GCKR* [9]. Furthermore, a recent study reported MAFLD-associated variants and the *PNPLA3* and *GATAD2A* genes in an East Asian population [11]. Similarly, a study conducted by Lin et al. on 904 lean individuals of Han Chinese descent revealed that rs738409 in *PNPLA3* is associated with fatty liver [12].

**Fig. 1:**
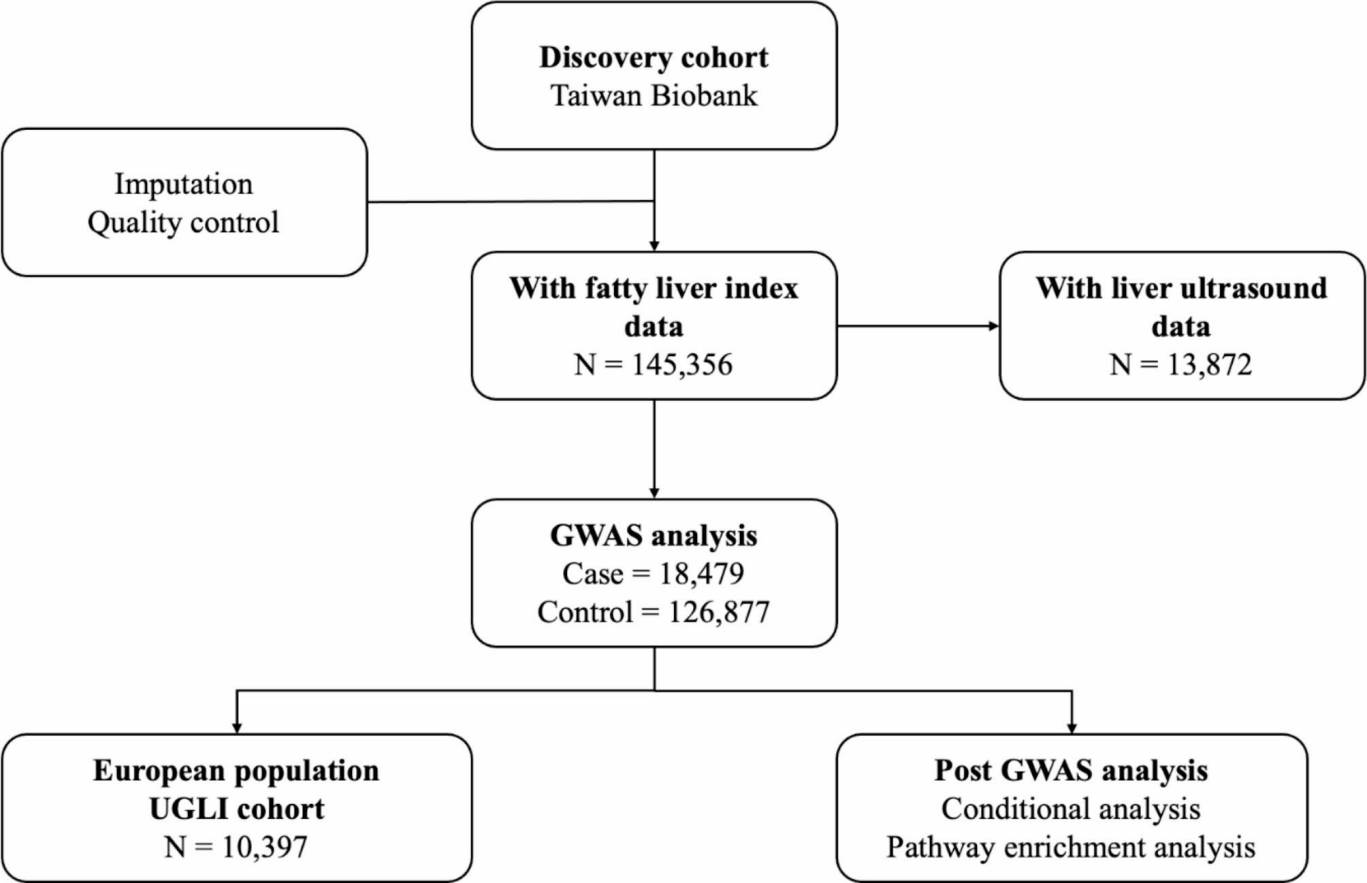
Flow diagram of the study design

In this study, we conducted a GWAS using a large population-based cohort using the FLI as a biomarker to identify individuals with FLI-defined fatty liver disease (FLI-defined FLD). Our results support the accuracy of FLI in detecting FLD and identifying the genetic risk factors associated with FLI-defined FLD in Taiwan Han Chinese and European populations (Fig. 1).

## Methods

### Study populations

We obtained our discovery cohort from the Taiwan Biobank (TWB), which collects genetic, clinical and lifestyle data from over 200,000 healthy individuals in Taiwan. Prior to their inclusion, participants provided informed consent, and approval was secured from the TWB, Academia Sinica, and the Institutional Review Board of Taipei Medical University (TMU-JIRB no. N201905005) for the execution of the study.

The TWB dataset includes various health information and testing results to assess various health conditions, including but not limited to participants’ self-reported questionnaires, physical examinations, genetic data, blood and urine samples, abdominal ultrasounds, bone mass density scans, and electrocardiograms [13]. Patients provide information on their clinical conditions, family history, dietary preferences, and alcohol consumption (defined as a minimum intake of 150 cc per week for 6 consecutive months).

Data for the European population were collected from the UMCG Genetics Lifelines Initiative (UGLI) cohort, an extension of the Lifelines Cohort Study, recruiting participants from the northern part of the Netherlands (https://www.lifelines-biobank.com/). The UGLI cohort released the genotyping data for approximately 38,500 (UGLI-1) and 28,000 (UGLI-2) participants; the data were generated via the Infinium Global Screening Array (GSA) chip (Illumina, CA, USA) and the FinnGen array (Thermo Fisher Scientific, MA, USA). The FLI GWAS was conducted with 10,398 independent European participants in UGLI-1 with complete laboratory test data for FLI calculation [14]. The summary statistics of the FLI GWAS can be obtained from Harvard Dataverse (10.7910/DVN/4YM1BG).

### Fatty liver characterization

The FLI score, which varies between 0 and 100, is used to assess the risk of developing FLD, with FLI ≥ 60 indicating a high likelihood of having a fatty liver condition. The FLI was calculated for each participant in the TWB cohort using the following formula [8]:

$$\:FLI\:=\frac{{e}^{y}}{1+{e}^{y}}\times\:100$$

where

$$\:y\:=\:{e}^{0.953\:\times\:\:ln\left(TG\right)\:+\:0.139\:\times\:\:BMI\:+\:0.718\:\times\:\:ln\:\left(GGT\right)\:+\:0.053\:\times\:\:WC\:-15.745}$$

TG = triglyceride (mg/dL).

BMI = body mass index (kg/m^2^).

GGT = gamma-glutamyl transferase (U/L).

WC = waist circumference (cm).

Individuals with missing data on body weight, height, WC, TG, or GGT were excluded from the analysis.

A subset of TWB participants eligible for FLI calculation also underwent abdominal ultrasound examinations. To assess the accuracy and reliability of the FLI as a diagnostic instrument for FLD, we conducted a chi-square test on the participants’ FLI values. They were then categorized based on the results of imaging evaluations completed by physicians. The control group included 9,978 individuals whose imaging results were indicative of “normal” or “no significant difference”, and the case group included 3,894 individuals with “mild,” “moderate,” or “severe” fatty liver conditions.

### Genotyping and quality control

The genomic DNA extracted from blood samples collected from the participants was genotyped by the National Centre for Genome Medicine (NCGM) at Academia Sinica using the TWB 2.0 chip. The dataset for whole-genome sequencing included 1,451 TWB subjects and 504 individuals from the East Asian (EAS) panel of the 1000 Genome Project, which served as a reference for imputation. Imputation procedures were conducted with IMPUTE (v2.3.1) software tools [15]. Quality control measures were applied using PLINK v2.0 [16] with specific criteria, including a call rate of over 98%, a minor allele frequency (MAF) greater than 0.05, adherence to Hardy‒Weinberg equilibrium (HWE) with a *p*-value exceeding 10^‒6^, and the exclusion of insertions and deletions.

Participants who exhibited discrepancies in sex between recorded and genotyped data possessed a call rate lower than 98% or who were identified as related (identity by descent (IBD) > 0.1875) were excluded from the research. Moreover, individuals whose heterozygosity levels surpassed the mean ± 3 standard deviations were also omitted to reduce the potential influence of DNA contamination or consanguinity. After implementing a series of quality control procedures and removing individuals with missing data for FLI calculation, a total of 145,356 participants were included in the GWAS analysis.

### Genome-wide association analysis

Logistic regression analysis was performed using the PLINK 2.0 software [16], with age, age^2^, sex, alcohol consumption and the first 10 principal components (PCs) included as covariates. A significance threshold of *P* < 5 × 10^− 8^ was utilized for genome-wide significance, whereas *P* < 10^− 5^ was considered indicative of genome-wide suggestive associations. Data visualization was achieved through the generation of Manhattan plots and quantile‒quantile (Q‒Q) plots via the CMplot R package. In this study, we utilized the Genome Reference Consortium Human Build 38 (GRCh38) to define single-nucleotide polymorphism (SNP) positions.

### Conditional analysis

To identify independent variants in our GWAS, we performed conditional analysis via GCTA-COJO with a stepwise model selection method on all significant SNPs from the GWAS results to isolate independent signals [17]. This approach identifies causal variants by isolating those that contribute independently to the observed associations. Variants were defined as independent if they met the following criteria: they were located within ± 10 Mb of the targeted region, showed low collinearity (cutoff of 0.9), and achieved genome-wide significance (*P* < 5 × 10^− 8^).

### Pathway enrichment analysis

For pathway enrichment analysis (PEA), we utilized all genes mapped from the significant SNPs identified in the GWAS. Significant SNPs were annotated via the Ensembl Variant Effect Predictor (VEP) [18] to map them to relevant genes, followed by cross-referencing with the GWAS catalog [19] to identify loci previously associated with NAFLD, total cholesterol (TC), TG, LDL-C, high-density lipoprotein cholesterol (HDL-C), metabolic traits, and BMI. We conducted PEA using WebGestalt [20], which focuses on Gene Ontology (GO) biological processes (BP) [21], Kyoto Encyclopedia of Genes and Genomes (KEGG) [22], Reactome [23], and WikiPathways [24]. Pathway significance was assessed using a false discovery rate (FDR) ≤ 0.05, and visualizations were generated via the ggplot2 package.

## Results

### Baseline characteristics of the participants

In this study, a total of 145,356 TWB participants were included in the discovery analysis (Fig. 1). The discovery cohort included 52,655 males and 92,701 females, with an overall average age of 49.42 years (Table 1). The case group included a higher proportion of male subjects (63.02%) compared to the control group (32.32%), aligned with the global prevalence of FLD by sex. TWB participants in the control group presented a healthier metabolic profile than those in the case group. The control group had a lower mean BMI (23.40 kg/m² vs. 30.23 kg/m²), mean TG level (99.71 mg/dL vs. 225.98 mg/dL), and mean LDL-C level (120.26 mg/dL vs. 125.15 mg/dL) than the case group. Additionally, the levels of liver enzymes, including serum glutamic-oxaloacetic transaminase (SGOT) (31.87 U/L vs. 23.51 U/L) and serum glutamic-pyruvic transaminase (SGPT) (43.03 U/L vs. 21.31 U/L), were elevated in the case group, suggesting potential liver inflammation or damage.

Among the study participants, 6.08% (8,840 individuals) reported alcohol consumption, with 27.8% belonging to the case group and 72.2% to the control group. Conversely, 93.92% (136,401 individuals) reported no alcohol consumption, with 11.7% in the case group and 88.3% in the control group.

A total of 145,356 TWB participants were genotyped and passed the quality control (QC) steps described in the Methods section, resulting in 18,479 cases and 126,877 controls according to the FLI cutoff. A subset of 13,872 participants with both FLI and ultrasound data were selected to evaluate the clinical accuracy of the FLI in detecting FLD. A GWAS was performed under an additive model via logistic regression adjusted for age, age^2^, sex, alcohol consumption, and the top 10 PCs. PEA was then conducted to explore the biological functions of the identified genes. An additional cohort with European samples was used to identify shared genetic determinants for FLI-defined FLD across different populations.

**Table 1:** Baseline characteristics of the TWB study cohort

Characteristics	All Subjects	Cases	Controls	*P* value
Number of samples	145,356	18,479	126,877	
Sex, men (%)	36.22	63.02	32.32	< 0.001
Age, yr	49.42 ± 11.38	48.67 ± 10.97	49.53 ± 11.43	< 0.001
Height, cm	162.09 ± 8.33	165.74 ± 8.91	161.55 ± 8.11	< 0.001
Weight, kg	64.06 ± 12.96	83.17 ± 13.03	61.29 ± 10.34	< 0.001
BMI, kg/m^2^	24.27 ± 3.85	30.23 ± 3.80	23.40 ± 2.99	< 0.001
Waistline, cm	83.40 ± 10.36	99.02 ± 8.72	81.12 ± 8.44	< 0.001
Total cholesterol, mg/dL	195.81 ± 37.07	203.22 ± 39.57	194.72 ± 36.56	< 0.001
Triglycerides, mg/dL	115.77 ± 94.27	225.98 ± 185.04	99.71 ± 56.29	< 0.001
HDL-C, mg/dL	54.85 ± 13.54	44.17 ± 9.71	56.41 ± 13.32	< 0.001
LDL-C, mg/dL	120.88 ± 31.90	125.15 ± 34.95	120.26 ± 31.39	< 0.001
Gamma GT, U/L	24.16 ± 31.52	54.24 ± 70.06	19.78 ± 16.50	< 0.001
SGOT, U/L	24.56 ± 12.95	31.87 ± 19.20	23.51 ± 11.39	< 0.001
SGPT, U/L	24.07 ± 21.32	43.03 ± 33.07	21.31 ± 17.37	< 0.001
FLI	25.92 ± 24.67	76.71 ± 10.93	18.53 ± 15.79	< 0.001
Alcohol consumption				
Yes	8,840	2,455	6,385	
No	136,401	16,010	120,391	

Note: The data are presented as the means ± sds

Among the discovery cohort, we found that individuals in the case group had a significantly increased risk of image confirmed FLD (OR = 4.43, *P* < 2.2 × 10⁻^16^). Additionally, participants with higher FLIs in the case group had an even greater risk of developing moderate to severe fatty liver (OR = 8.85, *P* < 2.2 × 10⁻^16^), indicating a strong association between the FLI and the severity of fatty liver.

### GWAS for FLI-defined fatty liver disease identifies novel and known loci

**Fig. 2:**
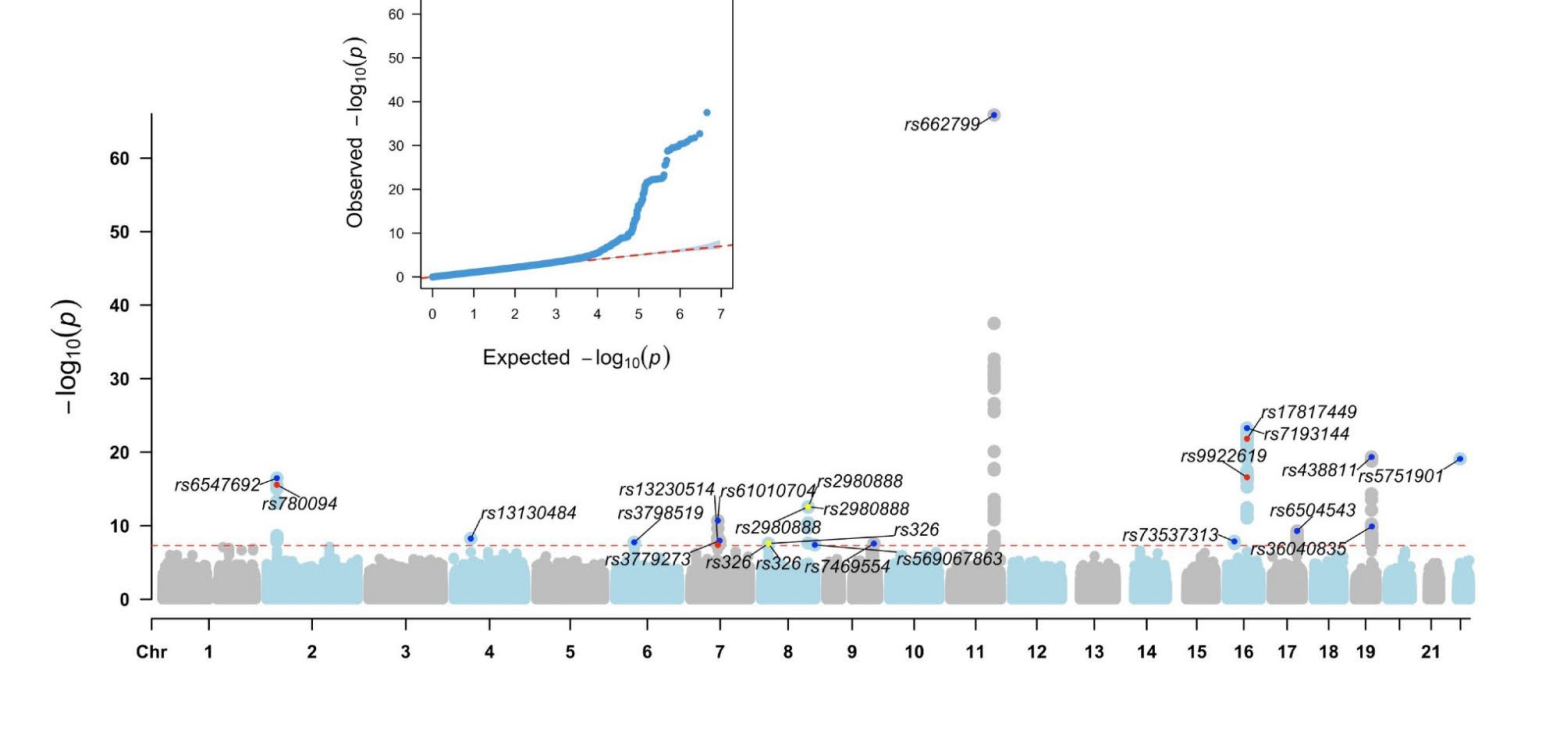
Manhattan plot of FLI-defined FLD in a Taiwanese population revealed 423 genome-wide loci. The red line corresponds to a genome-wide significance threshold of 5 × 10^− 8^. The variant indices in blue represent the independent risk variants of FLI-defined FLD; the variant indices in red represent reported variants associated with NAFLD; and the variants in yellow represent independent risk variants that were reported to be associated with NAFLD. The genomic inflation factor (λ_GC_) of the GWAS for FLI-defined FLD is 1.08

Data from the TWB were used to conduct a GWAS to investigate the genetic factors influencing FLI-defined FLD (Fig. 2). The variants that reached genome-wide significance (*P* < 5 × 10^− 8^) and suggestive significance (*P* < 1 × 10^− 5^) are listed in Additional Files 1 and 2, respectively. We identified 423 significant SNPs, with the most prominent peak observed on chromosomes 11q23.3 and 16q12.2. Most of the significant SNPs were located within intronic regions. Among the significant variants, 417 variants were novel, whereas 6 variants were reported by previous NAFLD GWAS according to the GWAS Catalog (assessed date 1/8/2024) (Table 2). Among the 31 genes annotated from these significant risk variants, eight were reported to be associated with NAFLD, including *BPTF*, *BUD13*, *FTO*, *GCKR*, *LPL*, *SIK3*, *TRIB1AL*, and *ZNF512.* In addition, we identified FLI-defined FLD risk variants that have previously been associated with lipid profiles that are highly correlated with fatty liver progression (Additional File 3–9).

To refine the identified genetic associations, we performed conditional and joint analysis on GWAS-significant SNPs, and mapping identified 16 independent loci mapped to 14 genes (Table 3). The associations align with metabolic traits and diseases previously reported in the GWAS Catalog; among the identified variants, rs6547692, rs662799, rs3779273, rs326, rs438811, and rs5751901 were associated with traits linked to lipid regulation, whereas rs61010704 in *MLXIPL* showed a notable association with metabolic syndrome and HDL. rs7193144 and rs13130484 were associated with obesity-related traits such as BMI, obesity, and related phenotypes. Another notable variant, rs2980888 in *TRIB1AL*, was strongly associated with NAFLD, corroborating its role in hepatic lipid metabolism.

The novel variants are as follows: the *RHPN1* intron variant rs569067863, the *LMX1B* intron variant rs7469554, the variant rs73537313 on chromosome 16, and the *BPTF* intron variant rs6504543. These novel loci present new avenues for research, as they lack previous associations in the existing databases, potentially offering insights into the underlying biological mechanisms and novel therapeutic targets for FLD.

**Table 2:** Known NAFLD variants identified in our GWAS for FLI-defined FLD

Chr	Pos	SNP	Function	Gene	RA	EA	EAF	BETA	*P*
2	27,518,370	rs780094	intron	GCKR	T	C	0.52	-0.09	2.70 × 10^− 16^
7	73,633,765	rs13230514	intergenic	-	A	G	0.75	-0.97	4.27 × 10^− 08^
8	19,961,928	rs326	intron	LPL	A	G	0.20	-0.08	2.62 × 10^− 08^
8	125,495,066	rs2980888	intron	TRIB1AL	T	C	0.72	-0.09	2.69 × 10^− 13^
16	53,779,455	rs17817449	intron	FTO	T	G	0.12	0.16	1.47 × 10^− 22^
16	53,797,859	rs9922619	intron	FTO	G	T	0.18	0.12	2.64 × 10^− 17^

Chr, chromosome; Pos, position; RA, reference allele; EA, effect allele; EAF, effect allele frequency; P, *P* value

**Table 3:** Significant SNPs of conditional independent analysis for FLI-defined FLD

Chr	SNP	RA	EA	Function	Gene	BETA	Z score	*P*	Associated traits*
2	rs6547692	G	A	intron	GCKR	-0.10	-8.43	3.41 × 10^− 17^	LDL-C, TG, TC
4	rs13130484	C	T	intergenic	-	0.08	5.83	5.60 × 10^− 09^	BMI, WC
6	rs3798519	A	C	intron	TFAP2B	0.08	5.63	1.80 × 10^− 08^	BMI, T2DM
7	rs61010704	A	G	intron	MLXIPL	-0.14	-6.70	2.03 × 10^− 11^	HDL-C
7	rs3779273	G	A	intron	MAGI2	-0.07	-5.72	1.07 × 10^− 08^	BMI
8	rs326	A	G	intron	LPL	-0.08	-5.57	2.62 × 10^− 08^	NAFLD
8	rs2980888	T	C	intron	TRIB1AL	-0.09	-7.31	2.69 × 10^− 13^	NAFLD
8	rs569067863	G	A	intron	RHPN1	1.21	5.49	4.03 × 10^− 08^	Novel
9	rs7469554	A	G	intron	LMX1B	-0.06	-5.56	2.68 × 10^− 08^	Novel
11	rs662799	G	A	upstream	ZPR1	-0.22	-17.24	1.39 × 10^− 66^	HDL-C, TG, TC
16	rs73537313	T	G	intron	-	-0.07	-5.68	1.38 × 10^− 08^	Novel
16	rs7193144	T	C	intron	FTO	0.17	10.10	5.24 × 10^− 24^	BMI
17	rs6504543	T	C	intron	BPTF	0.08	6.21	5.31 × 10^− 10^	Novel
19	rs438811	C	T	upstream	APOC1	0.13	9.17	4.60 × 10^− 20^	TG, TC
19	rs36040835	T	G	intron	QPCTL	0.15	6.43	1.26 × 10^− 10^	Novel
22	rs5751901	T	C	upstream	LRRC75B	0.11	9.11	8.16 × 10^− 20^	GGT

Chr, chromosome; RA, reference allele; EA, effect allele; P, *P*-value

*The variant-traits association were extracted from GWAS Catalog

### Shared risk variants for FLI-defined fatty liver disease among Taiwanese and European populations

To explore the shared genetic determinants of FLI-defined fatty liver across populations, we compared our GWAS-significant SNPs to those identified in the UGLI cohort. All the 50 shared significant risk variants (Table 4) were located on *FTO* and *ZPR1* gene. 

**Table 4:** The significant variants after validation via the UGLI FLI summary statistic

Chr	SNP	Function	Gene	Taiwan Biobank		UGLI	
				Beta	*P*	Beta	*P*
11	rs964184	3 UTR	ZPR1	-0.1560	6.73 × 10^− 30^	0.0377	3.35 × 10^− 13^
11	rs3741298	intron	ZPR1	-0.1028	2.60 × 10^− 18^	0.0318	1.98 × 10^− 12^
16	rs9937354	intron	FTO	0.1238	3.88 × 10^− 17^	0.0193	4.79 × 10^− 08^
16	rs9928094	intron	FTO	0.1238	3.88 × 10^− 17^	0.0193	4.79 × 10^− 08^
16	rs9930397	intron	FTO	0.1243	8.39 × 10^− 17^	0.0194	4.36 × 10^− 08^
16	rs9940278	intron	FTO	0.1239	3.97 × 10^− 17^	0.0194	4.36 × 10^− 08^
16	rs9939973	intron	FTO	0.1234	4.79 × 10^− 17^	0.0194	4.36 × 10^− 08^
16	rs9940646	intron	FTO	0.1234	4.79 × 10^− 17^	0.0194	4.36 × 10^− 08^
16	rs1421085	intron	FTO	0.1628	7.30 × 10^− 23^	0.0215	1.82 × 10^− 09^
16	rs11642015	intron	FTO	0.1608	2.90 × 10^− 22^	0.0215	1.82 × 10^− 09^
16	rs62048402	intron	FTO	0.1612	2.22 × 10^− 22^	0.0215	1.75 × 10^− 09^
16	rs1558902	intron	FTO	0.1617	1.65 × 10^− 22^	0.0214	2.05 × 10^− 09^
16	rs56094641	intron	FTO	0.1643	2.71 × 10^− 23^	0.0214	2.11 × 10^− 09^
16	rs55872725	intron	FTO	0.1634	2.04 × 10^− 22^	0.0217	1.30 × 10^− 09^
16	rs7187250	intron	FTO	0.1642	1.39 × 10^− 22^	0.0206	9.63 × 10^− 09^
16	rs7193144	intron	FTO	0.1686	5.24 × 10^− 24^	0.0205	1.06 × 10^− 08^
16	rs62033399	intron	FTO	0.1646	1.03 × 10^− 22^	0.0206	9.18 × 10^− 09^
16	rs62033400	intron	FTO	0.1670	1.74 × 10^− 23^	0.0204	1.22 × 10^− 08^
16	rs8063057	intron	FTO	0.1656	4.19 × 10^− 23^	0.0204	1.28 × 10^− 08^
16	rs17817449	intron	FTO	0.1638	1.47 × 10^− 22^	0.0206	9.90 × 10^− 09^
16	rs8043757	intron	FTO	0.1629	2.48 × 10^− 22^	0.0206	9.18 × 10^− 09^
16	rs9972653	intron	FTO	0.1659	3.41 × 10^− 23^	0.0207	7.50 × 10^− 09^
16	rs17817497	intron	FTO	0.1654	5.07 × 10^− 23^	0.0205	1.20 × 10^− 08^
16	rs8050136	intron	FTO	0.1634	1.91 × 10^− 22^	0.0205	1.13 × 10^− 08^
16	rs8051591	intron	FTO	0.1629	2.48 × 10^− 22^	0.0205	1.13 × 10^− 08^
16	rs9935401	intron	FTO	0.1634	1.77 × 10^− 22^	0.0205	1.13 × 10^− 08^
16	rs3751812	intron	FTO	0.1646	9.37 × 10^− 23^	0.0205	1.13 × 10^− 08^
16	rs3751814	intron	FTO	0.1656	4.64 × 10^− 23^	0.0205	1.19 × 10^− 08^
16	rs9936385	intron	FTO	0.1653	5.53 × 10^− 23^	0.0205	1.19 × 10^− 08^
16	rs9923233	intron	FTO	0.1660	3.84 × 10^− 23^	0.0205	1.19 × 10^− 08^
16	rs11075991	intron	FTO	0.1654	5.36 × 10^− 23^	0.0205	1.14 × 10^− 08^
16	rs11075992	intron	FTO	0.1652	5.95 × 10^− 23^	0.0205	1.14 × 10^− 08^
16	rs9926289	intron	FTO	0.1646	8.37 × 10^− 23^	0.0205	1.19 × 10^− 08^
16	rs9939609	intron	FTO	0.1654	5.25 × 10^− 23^	0.0205	1.19 × 10^− 08^
16	rs7206410	intron	FTO	0.1655	4.90 × 10^− 23^	0.0205	1.18 × 10^− 08^
16	rs7202116	intron	FTO	0.1658	3.86 × 10^− 23^	0.0205	1.18 × 10^− 08^
16	rs7202296	intron	FTO	0.1654	5.37 × 10^− 23^	0.0205	1.18 × 10^− 08^
16	rs66908032	intron	FTO	0.1659	3.95 × 10^− 23^	0.0205	1.18 × 10^− 08^
16	rs72803697	intron	FTO	0.1660	3.70 × 10^− 23^	0.0205	1.18 × 10^− 08^
16	rs62033403	intron	FTO	0.1658	4.09 × 10^− 23^	0.0205	1.18 × 10^− 08^
16	rs62033404	intron	FTO	0.1656	4.69 × 10^− 23^	0.0205	1.18 × 10^− 08^
16	rs62033405	intron	FTO	0.1648	8.00 × 10^− 23^	0.0204	1.33 × 10^− 08^
16	rs7206122	intron	FTO	0.1655	5.04 × 10^− 23^	0.0205	1.18 × 10^− 08^
16	rs79994966	intron	FTO	0.1609	3.30 × 10^− 20^	0.0204	1.25 × 10^− 08^
16	rs62033408	intron	FTO	0.1649	6.79 × 10^− 23^	0.0203	1.68 × 10^− 08^
16	rs17817964	intron	FTO	0.1571	5.70 × 10^− 23^	0.0203	1.47 × 10^− 08^
16	rs72805611	intron	FTO	0.1562	6.02 × 10^− 22^	0.0194	4.96 × 10^− 08^
16	rs72805612	intron	FTO	0.1560	6.86 × 10^− 22^	0.0194	4.96 × 10^− 08^
16	rs11075993	intron	FTO	0.1546	1.30 × 10^− 21^	0.0194	4.96 × 10^− 08^
16	rs12149574	intron	FTO	0.1540	1.67 × 10^− 21^	0.0194	4.70 × 10^− 08^

chr, chromosome; Pos, position; P, *P* value

### Pathway enrichment analysis for significant variants associated with fatty liver

**Fig. 3:**
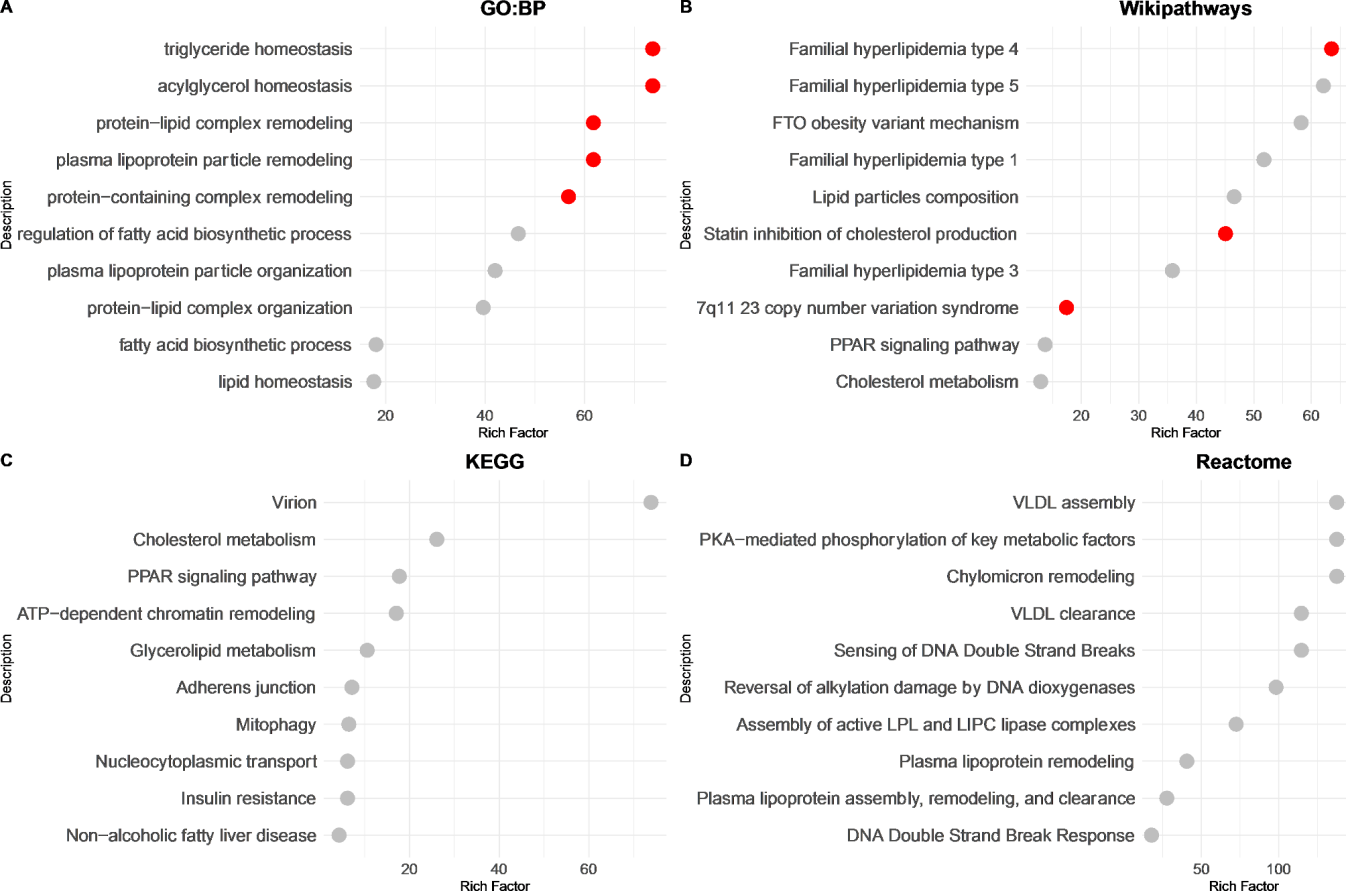
Pathway enrichment analysis of FLI-defined FLD risk genes. The top 10 enriched pathways from the **(A)** Gene Ontology Biological Process (GOBP), **(B)** WikiPathways, **(C)** KEGG and **(D)** Reactome databases. Pathways with FDR < 0.05 are displayed in red, while others are displayed in gray

A total of 31 FLI-defined FLD risk genes were included in the enrichment analysis, and the top 10 pathways from each database are shown in Fig. 3. The majority of the enriched GOBP pathways are related to fatty liver formation, such as TG homeostasis, acylglycerol homeostasis, and lipoprotein remodeling (Fig. 3A). The results from the Wikipathway analysis revealed that the FLI-defined FLD risk genes are involved in various types of familial hyperlipidemia, obesity, and cholesterol metabolism (Fig. 3B). KEGG pathway analysis revealed pathways related to NAFLD, cholesterol metabolism, and insulin resistance (Fig. 3C). The peroxisome proliferator-activated receptor (PPAR) signaling pathway was identified via both Wikipathway and KEGG analyses. Reactome analysis revealed pathways involved in TG synthesis, such as plasma lipoprotein assembly, remodeling, and clearance, as well as chylomicron remodeling and the assembly of active LPL and LIPC lipase complexes (Fig. 3D).

## Discussion

In this study, we conducted a GWAS for FLI-defined FLD using data from 145,356 Taiwanese individuals. Our GWAS identified 423 significant risk variants located in 31 genes, including 6 variants previously reported to be associated with NAFLD located on *GCKR*,* LPL*,* TRIB1AL*, and *FTO*. The primary aim of our discovery study was to identify genetic variants associated with FLD in a Taiwanese cohort using FLI as a diagnostic tool. The ultrasound results and evaluation verify the reliability of the FLI as an indicator of fatty liver, especially in moderate to severe cases. Although studies have shown that ultrasound has limitations in detecting mild fatty liver cases (< 20%) and individuals with a BMI over 40 kg/m^2^ [6], our study cohort, with a mean BMI of 24.27 kg/m^2^, aligns well within the ultrasound target range. This supports the utility of FLI as an alternative marker for FLD.

Our analysis strongly revealed that *FTO* and *ZPR1* variants are associated with FLD, which is consistent with prior studies linking these genes to metabolic traits and NAFLD. Variants in *ZPR1* have been shown to influence circulating TG and HDL-C levels through pathways related to lipid processing [25, 26]. As elevated TG levels are a direct contributor to FLD, *ZPR1* can be linked to NAFLD risk. Similarly, *FTO* is associated with hypercholesterolemia, which contributes to cardiovascular risk and is a well-established regulator of body weight, fat storage, and lipid metabolism, whereas variants in *FTO* have been associated with increased BMI and increased susceptibility to lipid and glucose dysregulation [27–30].

In addition to *ZPR1* and *FTO*, other genes identified in our GWAS were related to FLD. For example, *GCKR* variants are known to influence glucose and lipid metabolism, with elevated glucokinase activity promoting de novo hepatic lipogenesis and inhibiting fatty acid oxidation, mechanisms implicated in NAFLD development [31, 32]. Similarly, *LPL*, a key regulator of lipid metabolism, plays a critical role in TG hydrolysis and energy balance [33]. Dysregulation of these genes contributes to lipid accumulation and metabolic dysfunction, reinforcing their importance in fatty liver pathogenesis.

To validate these findings of genetic variants associated with FLD, we conducted a replication study in an external cohort of 10,398 subjects from the UGLI cohort in Europe and found FLD risk variants shared between Taiwanese and European populations in terms of *FTO* and *ZPR1*. These associations were validated by another study consisting of a European cohort. However, population-specific differences were observed; for example, the strongest association in Europeans was with rs55872725, whereas in Taiwanese participants, it was with rs7193144. Despite these differences, both variants demonstrated a positive correlation with FLD, supporting their potential role in the development of the condition. Our validation study emphasized the population-specific and common genetic factors that contribute to FLD across different ancestries.

In addition to identifying individual gene variants, our PEA highlighted the importance of the PPAR signaling pathway and lipid metabolic pathways involving LDL-C, HDL-C, and TG in the progression of FLD. Although the results from the Wikipathway analysis and KEGG pathway analysis did not reach statistical significance, the identification of NAFLD substantiated the risk genes as strong signals for FLD. PPAR signaling regulates key processes such as adipogenesis, insulin resistance, and inflammation, which are central to NAFLD pathogenesis, and has been implicated in metabolic syndrome in previous GWASs of the Taiwanese population [34, 35]. These findings provide valuable insights into the genetic architecture of NAFLD and offer potential targets for further research and therapeutic interventions aimed at mitigating FLD and its metabolic complications.

Our findings also revealed enrichment in pathways related to lipid homeostasis and metabolism. TG accumulation, a hallmark of NAFLD, is believed to contribute to hepatocyte injury through mechanisms involving insulin resistance and disrupted glucose homeostasis [36]. Lipoprotein remodeling and the clearance of plasma lipoproteins, along with chylomicron remodeling, appear to play a role in lipid transport and processing, which can exacerbate fatty liver when disrupted [37]. Additionally, with dyslipidemia being a key factor in hepatic lipid accumulation, pathways linked to cholesterol metabolism being enriched underscore their involvement in the progression from simple FLD to more severe forms of NAFLD [29]. Regarding disease severity, we compared the genetic variants between fatty liver and liver fibrosis, but none of the variants were identical [38].

This study has several limitations. First, the detailed clinical information, such as diagnostic codes, liver biopsy results, or MRI data, is lacking, which may limit the ability to accurately reflect the real-world progression of FLD. Second, the FLI calculation was performed based on the timing of the blood samples collection, which may not fully account for individual variations. Relying on one-time measurements could therefore lead to misleading conclusions. Longitudinal data or more detailed clinical information would be necessary to gain a more comprehensive understanding of an individual’s health status.

## Conclusion

Our study highlights the genetic heterogeneity underlying FLI-defined FLD and emphasizes the relevance of key genetic variants across different populations. We not only confirmed previously identified genetic risk factors but also discovered novel variants associated with FLD. The findings of shared risk variants in the *FTO* and *ZPR1* genes suggest that common genetic mechanisms may influence FLD susceptibility. Moreover, the involvement of PPAR signaling and the plasma lipoprotein pathway in fatty liver progression highlights promising targets for therapeutic intervention. These findings also support the potential of the FLI as a reliable clinical tool for diagnosing FLD, but further research is still needed to clarify the role of these pathways in disease development and to explore the utility of the FLI in broader clinical applications.

## Electronic supplementary material

Below is the link to the electronic supplementary material.

**Additional File 1.** Genome-wide significant SNPs from FLI GWASs in the Taiwanese population. **Additional File 2**. Genome-wide suggestive SNPs from FLI GWAS in the Taiwanese population. **Additional File 3.** Genome-wide significant SNPs from the FLI GWAS previously reported for BMI in the GWAS Catalog. **Additional File 4.** Genome-wide significant SNPs from the FLI GWAS previously reported for HDL-C in the GWAS Catalog. **Additional File 5.** Genome-wide significant SNPs from the FLI GWAS previously reported for LDL-C in the GWAS Catalog. **Additional File 6.** Genome-wide significant SNPs from the FLI GWAS previously reported for metabolic disease in the GWAS Catalog. **Additional File 7.** Genome-wide significant SNPs from FLI GWASs previously reported for TC in the GWAS Catalog. **Additional File 8.** Genome-wide significant SNPs from the FLI GWAS previously reported for TG in the GWAS Catalog. **Additional File 9.** Genome-wide significant SNPs from the FLI GWAS previously reported for WC in the GWAS Catalog

## Data Availability

The datasets used and/or analyzed during the current study are available from the corresponding author upon reasonable request. The raw TWB dataset is available at https://www.twbiobank.org.tw/. The summary statistics of the FLI GWAS using the UGLI cohort can be accessed at 10.7910/DVN/4YM1BG.

## Abbreviations

ALD: Alcoholic-related liver disease
BMI: Body mass index
CT: Computed tomography
FDR: False discovery rate
FLD: Fatty (steatotic) liver disease
FLI: Fatty liver index
GGT: Gamma-glutamyl transferase
GWAS: Genome-wide association study
HDL-C: High-density lipoprotein cholesterol
KEGG: Kyoto Encyclopedia of Genes and Genomes
LDL-C: Low-density lipoprotein cholesterol
MAF: Minor allele frequency
MAFLD: Metabolic dysfunction-associated fatty liver disease
MASLD: Metabolic dysfunction-associated steatotic liver disease
MetALD: Metabolic dysfunction-and alcohol-associated liver disease
MRI: Magnetic resonance imaging
NAFLD: Metabolic dysfunction-and alcohol-associated liver disease
PEA: Pathway enrichment analysis
PPAR: Peroxisome proliferator-activated receptor
SGOT: Serum glutamic-oxaloacetic transaminase
SGPT: Serum glutamic-pyruvic transaminase
SNP: Single-nucleotide polymorphism
T2DM: Type 2 diabetes mellitus
TC: Total cholesterol
TG: Triglyceride
TWB: Taiwan Biobank
WC: Waist circumference
